# Mechanisms Underlying the Biphasic Effects of Hydrogen Sulfide in Rat Intrapulmonary Arteries

**DOI:** 10.3390/biomedicines14092043

**Published:** 2026-09-11

**Authors:** Agilė Tunaitytė, Elif Alan, Arnas Aleknavičius, Silvijus Abramavičius, Ulf Simonsen, Edgaras Stankevičius

**Affiliations:** 1 Preclinical Research Laboratory for Medicinal Products, Institute of Cardiology, Lithuanian University of Health Sciences, 50162 Kaunas, Lithuania; arnas.aleknavicius@stud.lsmu.lt (A.A.); edgaras.stankevicius@lsmu.lt (E.S.); 2 Institute of Physiology and Pharmacology, Lithuanian University of Health Sciences, 44307 Kaunas, Lithuania; silvijus.abramavicius@lsmu.lt; 3 Department of Biomedicine, Pulmonary and Cardiovascular Pharmacology, Aarhus University, Høgh-Guldbergsgade 10, 8000 Aarhus, Denmark; elifaa@biomed.au.dk (E.A.); us@biomed.au.dk (U.S.); 4 Department of Pharmacology, Faculty of Pharmacy, Ege University, 35040 Izmir, Türkiye

**Keywords:** calcium, nitric oxide, hydrogen sulfide, pulmonary arteries, vascular tone, oxidative stress

## Abstract

**Background/Objectives**: Hydrogen sulfide (H_2_S) is an important regulator of pulmonary vascular tone; however, the mechanisms underlying its biphasic contractile and relaxant effects, as well as the distinct contributions of endogenous and exogenous H_2_S to nitric oxide (NO) signaling, remain incompletely understood. This study investigated the roles of endogenous and exogenous H_2_S in regulating pulmonary vascular responses under physiological and oxidative stress conditions. **Methods**: Rat intrapulmonary arteries were studied using wire myography and simultaneous measurements of vascular force and intracellular Ca^2+^. Pharmacological inhibitors were used to examine the contribution of endogenous H_2_S synthesis, NO synthase, soluble guanylate cyclase (sGC), K_ATP_ channels, and oxidative stress to vascular responses. **Results**: Sodium hydrogen sulfide (NaHS) produced biphasic concentration-dependent responses, with contraction at low concentrations followed by relaxation at higher concentrations. Simultaneous measurements of vascular force and intracellular Ca^2+^ showed that the initial contractile response to low concentrations of NaHS was not accompanied by a clear increase in intracellular Ca^2+^, whereas relaxation developed despite maintained or increasing intracellular Ca^2+^, indicating that NaHS-induced relaxation cannot be explained solely by reduced intracellular Ca^2+^ levels. Inhibition of endogenous H_2_S synthesis reduced vascular responsiveness to the NO donor sodium nitroprusside, whereas relaxation induced by exogenous NaHS was largely preserved after inhibition of NO synthase or sGC, indicating distinct roles of endogenous and exogenous H_2_S. Oxidative stress impaired acetylcholine- and riociguat-induced relaxation, while NaHS partially restored endothelial function. **Conclusions**: Endogenous and exogenous H_2_S regulate pulmonary vascular tone through distinct mechanisms. Endogenous H_2_S supports vascular responsiveness to NO, whereas exogenous H_2_S induces relaxation largely independently of NO–sGC signaling. Furthermore, NaHS-induced relaxation occurs despite maintained intracellular Ca^2+^, suggesting an important contribution of Ca^2+^-independent mechanisms, and partially preserves endothelial function under oxidative stress.

## 1. Introduction

Pulmonary vascular tone is closely regulated by a complex interaction of endothelial mediators that manage smooth muscle contraction and relaxation. Among them, nitric oxide (NO) is a key endothelium-derived vasodilator in pulmonary arteries. It activates sGC and cyclic guanosine monophosphate (cGMP) signaling in vascular smooth muscle cells (VSMCs), thereby helping maintain low pulmonary vascular resistance under normal conditions. Deficiencies in NO production or signaling are critical in the development of pulmonary vascular diseases, such as pulmonary hypertension (PH), in which endothelial dysfunction and oxidative stress reduce the ability of blood vessels to relax and dilate [1].

PH is defined as a resting mean pulmonary artery pressure of 20 mm Hg or above. Patients with PH suffer from dyspnea, impaired exercise capacity, and premature death. Patients with PH have a mortality rate reaching 50% at five years. PH alters the physiology of the pulmonary vasculature via remodeling involving endothelial cells, smooth muscle cells, and fibroblasts. The intima, smooth muscle cells, and adventitial fibroblasts develop resistance to apoptosis [2], proliferate, and narrow the lumen of pulmonary arteries, thus causing PH. State-of-the-art therapies, such as prostaglandins, phosphodiesterase-5 inhibitors, endothelin receptor antagonists, and sGC stimulators, improve symptoms of PH but have limited impact on mortality [3,4].

Hydrogen sulfide (H_2_S), known as the third gasotransmitter, plays a crucial role in both the normal functioning and pathological conditions of the pulmonary vasculature. It is formed from L-cysteine via enzymatic pathways involving cystathionine γ-lyase (CSE), cystathionine β-synthase (CBS), and 3-mercaptopyruvate sulfurtransferase (3-MST) in vascular tissue. H_2_S has several effects on the cardiovascular system, including vasodilation, antioxidant activities, and cytoprotective actions. It also helps maintain vascular balance by regulating ion channels and redox balance [5,6].

The pulmonary circulation appears highly sensitive to H_2_S signaling. H_2_S has also been proposed to function as an oxygen (O_2_) sensor and transducer, contributing to vascular responses to hypoxia in the pulmonary circulation [7,8]. H_2_S donors cause pulmonary vasodilation in experimental models of pulmonary hypertension and can lower pulmonary artery pressure under experimental conditions [9]. However, in PH, the natural production and signaling of H_2_S are reduced, suggesting that dysregulation of H_2_S pathways may contribute to disease progression [6,9]. Importantly, H_2_S interacts with NO signaling in several ways. Studies in systemic arteries have shown that H_2_S can improve endothelial NO synthase (eNOS) activity, stabilize sGC in its NO-responsive reduced form, and inhibit phosphodiesterase activity [10]. This interaction promotes NO–cGMP signaling [10,11]. On the other hand, NO can affect endogenous H_2_S production, showing a two-way regulatory relationship [12].

In addition to its interactions with NO signaling, H_2_S has been shown to regulate vascular tone by modulating potassium channels, particularly ATP-sensitive potassium (K_ATP_) channels. Activation of these channels promotes membrane hyperpolarization and vascular smooth muscle relaxation and is considered an important mechanism underlying H_2_S-induced vasodilation [13,14].

Oxidative stress impairs regulation of the pulmonary vascular system by reducing NO availability and sGC responsiveness. H_2_S has been shown to have antioxidant effects. It scavenges reactive oxygen species (ROS), improves glutathione-dependent redox capacity, and maintains NO bioavailability. This suggests that H_2_S may help protect NO–sGC signaling under oxidative conditions [15,16].

Despite growing understanding, the exact role of endogenous H_2_S in NO-mediated relaxation in pulmonary arteries and how H_2_S affects sGC function under oxidative stress remain incompletely understood. Therefore, this study aimed to explore the relationship between endogenous and exogenous H_2_S and NO signaling in the relaxation of rat intrapulmonary arteries. In particular, we examined whether endogenous H_2_S contributes to NO-mediated relaxation, how sGC signaling is involved, how these interactions are affected by oxidative stress, and the contribution of K_ATP_ channels to H_2_S-mediated responses. To our knowledge, this is the first study to investigate the contribution of endogenous H_2_S to NO-mediated relaxation in rat intrapulmonary arteries and to examine the roles of sGC signaling, K_ATP_ channels, and oxidative stress in H_2_S-mediated vascular responses.

## 2. Materials and Methods

### 2.1. Animal Handling

The study adhered to the ethical standards outlined in the Guide for the Care and Use of Laboratory Animals, as published by the United States National Institutes of Health (NIH Publication No. 85–23, revised 1996), and conformed to the ARRIVE guidelines [17]. Adult male Wistar rats aged 12–14 weeks were humanely euthanized through decapitation followed by exsanguination. The experimental protocol received approval from the Lithuanian State Food and Veterinary Service (permission no. G2-288 2024-10-02). The experimental protocol also received approval from the Danish Animal Experiments Inspectorate (permission no. 2017-15-0201-01351).

### 2.2. Tissue Preparation and Wire Myography

Rat intrapulmonary arteries (~300 µm internal diameter) were carefully dissected from the lungs and mounted on two 40-µm steel wires in microvascular myographs (Danish Myotechnology, Aarhus, Denmark) for isometric tension recordings, as previously described [18]. Vessel normalization was performed to determine the lumen diameter (d100) corresponding to a transmural pressure of 23 mmHg, after which the vessels were set to 90% of d100. Arterial segments were precontracted with U46619 (3 × 10^−7^ M). In experiments assessing relaxation responses, cumulative concentration–response curves were generated using ACh (10^−10^–10^−5^ M) followed by SNP (10^−10^–3 × 10^−5^ M).

### 2.3. Substances Used in the Experiments

All chemicals were bought from Sigma-Aldrich (St. Louis, MO, USA). The drugs were prepared as stock solutions in distilled water (ACh, SNP, PPG, tempol, NaHS, L-cysteine, α-ketoglutarate, aspartate), HCl (L-NOARG), or dimethyl sulfoxide (DMSO) (U46619, glibenclamide, AOA, indomethacin, ODQ, pyrogallol, riociguat). The stock solutions were further diluted with distilled water to ensure that the final organ bath concentration of the solvent DMSO was below 0.01%.

### 2.4. Experimental Protocol

#### 2.4.1. Role of Endothelium, NO, K_ATP_ Channels and Intracellular Calcium in NaHS Responses

We examined the roles of the endothelium, inhibition of NO synthase, and blockade of K_ATP_ channels in relaxation induced by NaHS, an H_2_S donor, and sodium nitroprusside (SNP). The vessels were contracted with U46619 (3 × 10^−7^ M) and relaxed with NaHS (1 × 10^−7^–1 × 10^−2^ M) or SNP (1 × 10^−12^–1 × 10^−6^ M) in the presence/absence of a NO synthase inhibitor, Nω-nitro-L-arginine (L-NOARG, 10^−4^ M, 30 min). To assess endothelial involvement, the endothelium was removed by gently rubbing the luminal surface of the vessel. Successful removal of the endothelium was confirmed by the absence of acetylcholine (ACh)-induced relaxation. The U46619-contracted vessels were then relaxed with NaHS or SNP. To test the role of K_ATP_ channels, vessels were incubated with the K_ATP_ channel blocker, glibenclamide (10^−6^ M, 30 min), and contracted with U46619, followed by relaxation induced by NaHS or SNP.

To further characterize the responses to NaHS, simultaneous measurements of vascular tension and intracellular calcium were performed using the method described by Hedegaard et al. [19]. Small intrapulmonary arteries were loaded with the calcium-sensitive fluorescent dye Fura-2 acetoxymethyl ester (Fura-2 AM) and mounted in a wire myograph equipped for simultaneous force and fluorescence measurements. Arteries were precontracted with either U46619 (3 × 10^−7^ M) or potasium–rich physiological saline solution (KPSS) and exposed to cumulative concentrations of NaHS (1 × 10^−7^–1 × 10^−2^ M). Intracellular calcium was assessed as the Fura-2 fluorescence ratio (340/380 nm) and recorded simultaneously with changes in vascular tension.

#### 2.4.2. Role of Endogenous H_2_S in NO-Mediated Relaxation

Small intrapulmonary arteries were incubated with PPG (10 mM, 30 min), an inhibitor of CSE; PPG + aminooxyacetic acid (AOA, 10 mM, 30 min), an inhibitor of CBS; L-NOARG (10^−4^ M, 30 min), an inhibitor of nitric oxide synthase + indomethacin (3 μM, 30 min), an inhibitor of cyclooxygenase. The combination of L-NOARG and indomethacin was used to inhibit both NO- and cyclooxygenase-derived signaling pathways and thereby assess the contribution of endogenous H_2_S in the absence of these vasoactive influences. Arteries were also incubated with PPG + L-NOARG + indomethacin and then contracted with U46619 (3 × 10^−7^ M) and relaxed with ACh (1 × 10^−9^–1 × 10^−4^ M) or SNP (1 × 10^−12^–1 × 10^−6^ M).

#### 2.4.3. Role of sGC in NO- and H_2_S-Mediated Relaxation

We examined the role of sGC using the selective inhibitor 1H-[1,2,4]oxadiazolo[4,3-a]quinoxalin-1-one (ODQ, 3 × 10^−6^ M, 20 min) in NaHS- and SNP-induced relaxation.

#### 2.4.4. Role of Supplying the Substrate for CSE and 3-MST

The intrapulmonary vessels were contracted with U46619 (3 × 10^−7^ M) and relaxed with L-cysteine (1 × 10^−6^–1 × 10^−2^ M) in the presence/absence of PPG (10 mM, 30 min) or α-ketoglutarate (1 × 10^−6^–1 × 10^−2^ M) in the presence/absence of aspartate (10 mM, 30 min).

#### 2.4.5. Role of Oxidation of sGC on Relaxation Responses

To examine the role of oxidation of sGC, the vessels were incubated with pyrogallol (100 μM, 20 min) to generate superoxide and test the effect on ACh (1 × 10^−9^–1 × 10^−4^ M), SNP (1 × 10^−12^–1 × 10^−6^ M), and riociguat (1 × 10^−9^–1 × 10^−4^ M), an sGC stimulator. To test whether NaHS and tempol can modify vascular responses under these conditions, vessels were also incubated with NaHS (50 µM, 30 min) or tempol, a free radical scavenger (1 × 10^−4^ M, 20 min), after pyrogallol incubation. They were then contracted with U46619 (3 × 10^−7^ M) and relaxed with ACh, SNP, or riociguat. The concentration of NaHS (50 µM) was selected based on previous studies demonstrating that low micromolar concentrations of H_2_S donors restore redox-sensitive sGC signaling and improve vascular responsiveness under conditions associated with impaired H_2_S bioavailability and sGC oxidation [20].

### 2.5. Data and Statistical Analysis

The data are presented as mean ± SEM; *p* < 0.05 was considered statistically significant, and n represents the number of animals. Concentration–response curves were analyzed using two-way ANOVA to evaluate the effects of treatment, concentration, and interaction. Where a significant interaction or treatment effect was identified, Tukey’s post hoc test was used for multiple comparisons to determine the concentrations at which significant differences occurred. All statistical analyses were conducted using GraphPad Prism version 10.1.0 (GraphPad Software, San Diego, CA, USA). The results of the two-way ANOVA analyses, including treatment effects, concentration effects, and treatment × concentration interactions, are summarized in Supplementary Table S2. Each experiment was performed 5–6 times using vessels harvested from different animals. Sample sizes were determined based on prior laboratory experience and published studies that employed wire myography in rat pulmonary arteries. To further support this approach, a power analysis was conducted using G*Power (version 3.1.9.7) and preliminary vascular reactivity data. The analysis showed that a sample size of six observations would be sufficient to achieve 80% power at an α level of 0.05, consistent with the sample size used in this study.

## 3. Results

### 3.1. Mechanisms Underlying NaHS-Induced Biphasic Responses in Rat Intrapulmonary Arteries

**Figure 1:**
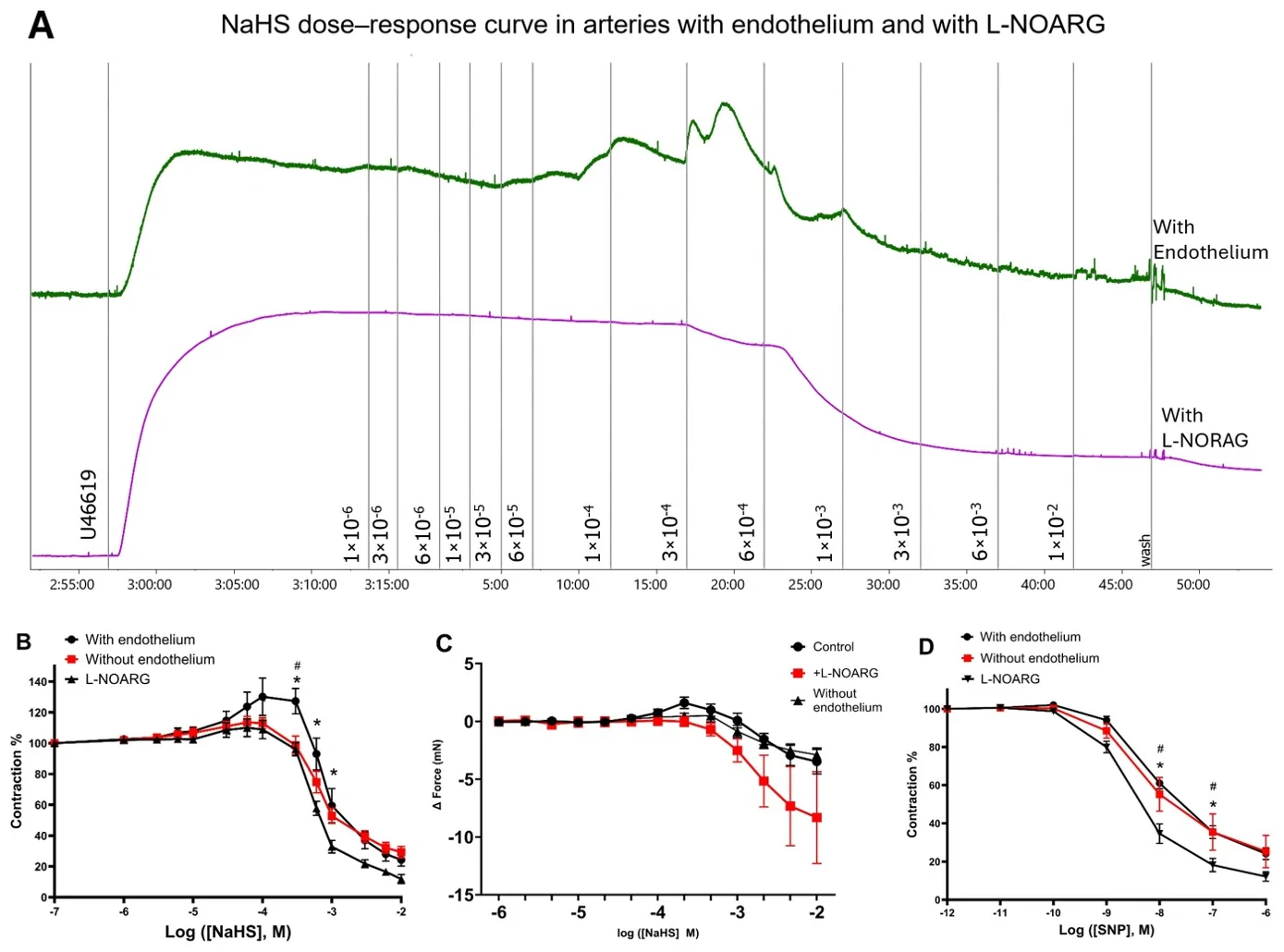
Endothelium- and NO-dependent modulation of NaHS-induced biphasic responses in rat intrapulmonary arteries. (**A**) NaHS-induced relaxation in rat pulmonary arteries, original trace data in arteries with endothelium and in the presence of L-NOARG. (**B**) NaHS- and (**D**) SNP-induced relaxation in rat intrapulmonary arteries with or without endothelium, and with endothelium in the presence of an inhibitor of nitric oxide synthase (L-NOARG, 10^−4^ M, 30 min), expressed as a percentage of precontraction. (**C**) NaHS-induced force responses: data are expressed as Δ force (mN), calculated as the force after each NaHS concentration minus the U46619-induced precontraction. The results are expressed as mean ± SEM (n = 6). Two-way ANOVA was used to evaluate the effects of treatment and concentration, and Tukey’s post hoc test was used for comparisons: * *p* < 0.05 versus arteries with endothelium. # *p* < 0.05 versus arteries without endothelium. Results of the two-way ANOVA analyses are presented in Supplementary Table S2.

**Figure 2:**
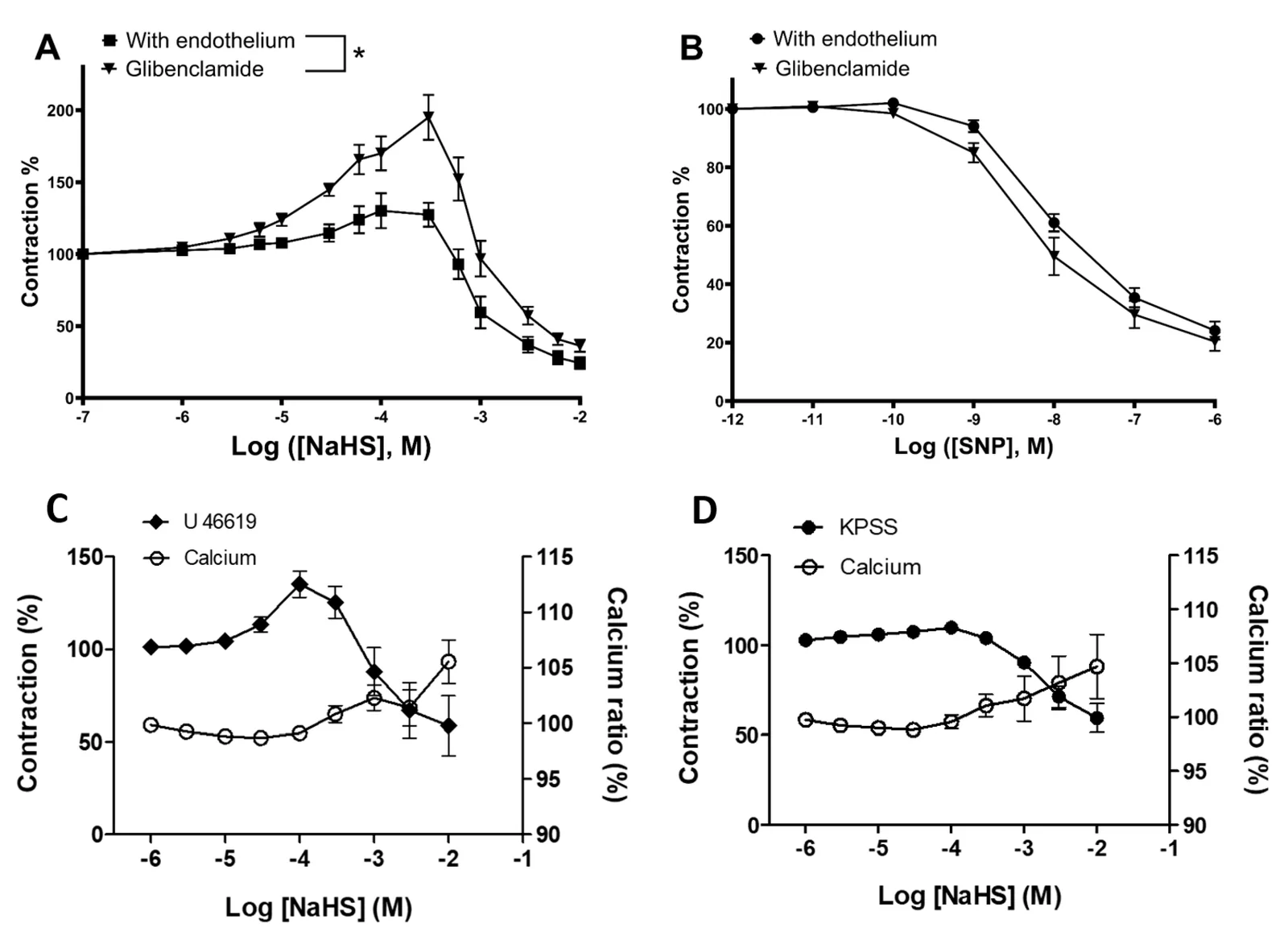
Role of K_ATP_ channels and intracellular Ca^2+^ in NaHS-induced responses in rat intrapulmonary arteries. (**A**) Concentration–response curves to NaHS in endothelium-intact intrapulmonary arteries in the absence and presence of glibenclamide (10^−6^ M, 30 min). (**B**) Concentration–response curves to SNP in the absence and presence of glibenclamide. (**C**,**D**) Simultaneous recordings of force and intracellular calcium during cumulative addition of NaHS in arteries precontracted with U46619 (**C**) or KPSS (**D**). Intracellular calcium is expressed as the normalized Fura-2 fluorescence ratio (340/380). Data are presented as mean ± SEM of n = 6 experiments. Two-way ANOVA was used to evaluate the effects of treatment and concentration in (**A**,**B**). * *p* < 0.05 versus the absence of glibenclamide. Results of the two-way ANOVA are presented in Supplementary Table S2.

NaHS induced a biphasic concentration-dependent response, characterized by contraction at lower concentrations followed by relaxation at higher concentrations in intrapulmonary arteries isolated from rats. The NaHS contractions were reduced when expressed as a percentage of precontraction following endothelial cell removal (Figure 1A,B), while relaxation induced by NaHS and the NO donor, SNP, was unaltered (Figure 1A,B,D). SNP normally acts as a NO donor, releasing NO that activates sGC and increases cGMP levels in smooth muscle cells, leading to relaxation [21]. As precontraction levels varied across experimental conditions, relaxation responses are expressed as a percentage of the precontraction level to minimize the impact of these differences. Analysis of force changes relative to the U46619-induced precontraction showed that the contractile component of the NaHS response was largely preserved, whereas the most pronounced differences were observed during the relaxant phase (Figure 1C). Incubation with the NO synthase inhibitor L-NOARG also reduced the apparent contractile response and increased the relaxant response induced by NaHS (Figure 1A–C). SNP-induced relaxation was also increased in the presence of L-NOARG (Figure 1D). In the presence of the K_ATP_ channel blocker glibenclamide, NaHS-induced contractions increased (Figure 2A), while SNP-induced relaxation was unaltered (Figure 2B). Simultaneous measurements of vascular force and intracellular Ca^2+^ showed that the initial contractile response to low concentrations of NaHS (30–100 μM) was not associated with a clear increase in intracellular Ca^2+^. As NaHS concentrations increased, relaxation developed both in U46619 and KPSS-contracted preparations despite a progressive increase in intracellular Ca^2+^, indicating that relaxation could not be explained by reduced intracellular Ca^2+^ levels (Figure 2C,D).

### 3.2. Contribution of Endogenous H_2_S to Endothelium-Dependent and NO-Mediated Relaxation

**Figure 3:**
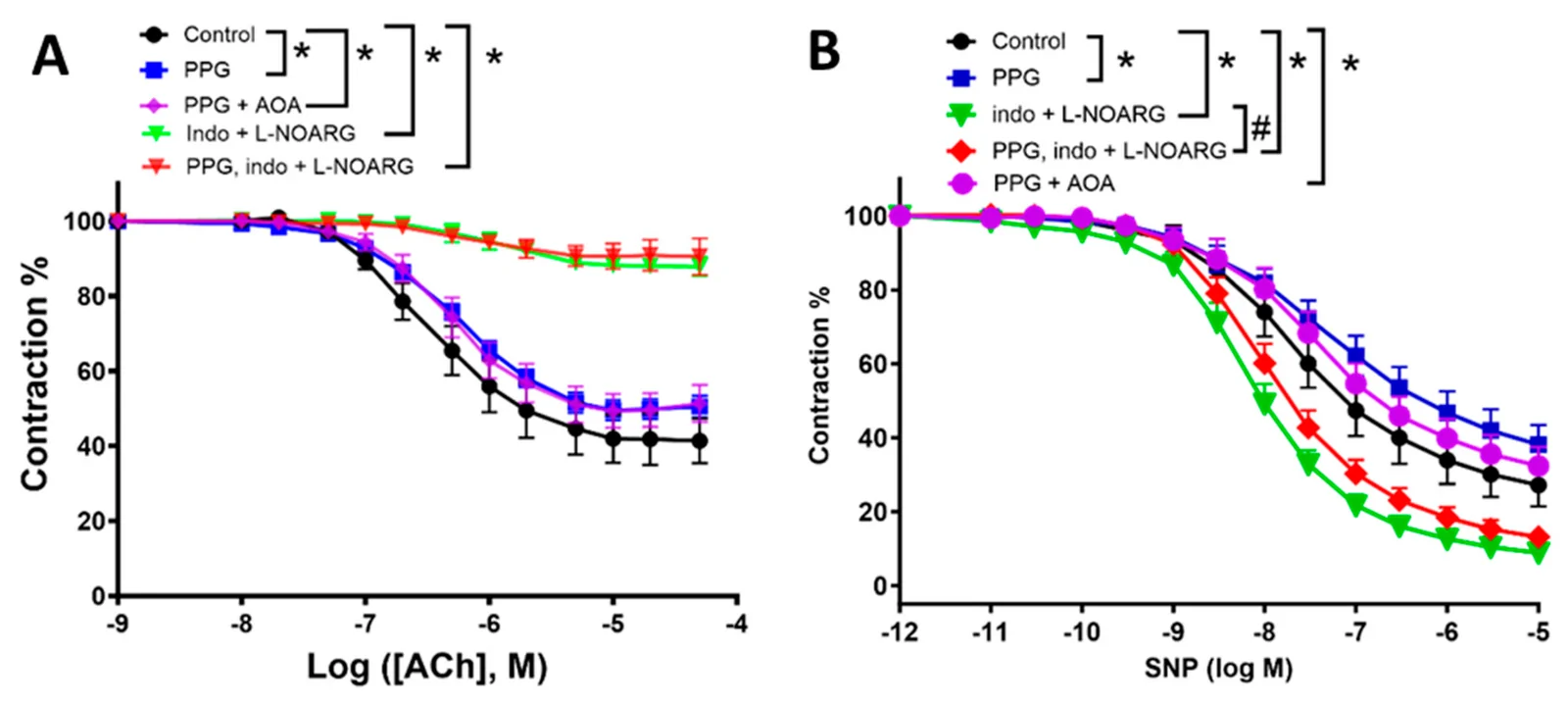
Contribution of endogenous H_2_S to endothelium-dependent and NO-mediated relaxation in rat intrapulmonary arteries. (**A**) ACh and (**B**) SNP relaxation in rat intrapulmonary arteries. The arteries were incubated with an inhibitor of CSE (PPG, 10 mM, 30 min), PPG plus an inhibitor of CBS (AOA, 10 mM, 30 min), an inhibitor of nitric oxide synthase (L-NOARG, 10^−4^ M, 30 min) plus an inhibitor of cyclooxygenase (indomethacin, 3 μM, 30 min), or a combination of PPG, L-NOARG, and indomethacin. After that, the preparations were contracted with U46619 and relaxed by increasing concentrations of ACh and SNP. The results are expressed as mean ± SEM of n = 6. Two-way ANOVA was used to evaluate the effects of treatment and concentration: * *p* < 0.05 versus arteries with endothelium, # *p* < 0.05 indo + L-NOARG versus PPG, indo + L-NOARG. Results of the two-way ANOVA analyses are presented in Supplementary Table S2.

To investigate whether endogenous H_2_S is involved in ACh relaxation of rat pulmonary arteries, the preparations were incubated with an inhibitor of CSE (PPG) or an inhibitor of CSE and CBS (PPG plus AOA). Incubation with PPG reduced ACh and SNP relaxation in rat pulmonary arteries (Figure 3). There was no additional effect of incubation with AOA on ACh- or SNP-induced relaxation. Incubation with L-NOARG and an inhibitor of cyclooxygenase, indomethacin, abolished ACh relaxation and enhanced SNP relaxation (Figure 3). In the presence of L-NOARG and indomethacin, there was no additional effect of PPG on ACh relaxation, suggesting that the effect of PPG is permissive and requires the presence of NO (Figure 3A). In contrast, in the presence of L-NOARG and indomethacin, the addition of PPG reduced SNP relaxation (Figure 3B). These findings indicate that inhibition of endogenous H_2_S synthesis reduced vascular responses to both endogenous and exogenous NO in rat intrapulmonary arteries.

### 3.3. Role of Soluble Guanylate Cyclase in NaHS- and SNP-Induced Responses

**Figure 4:**
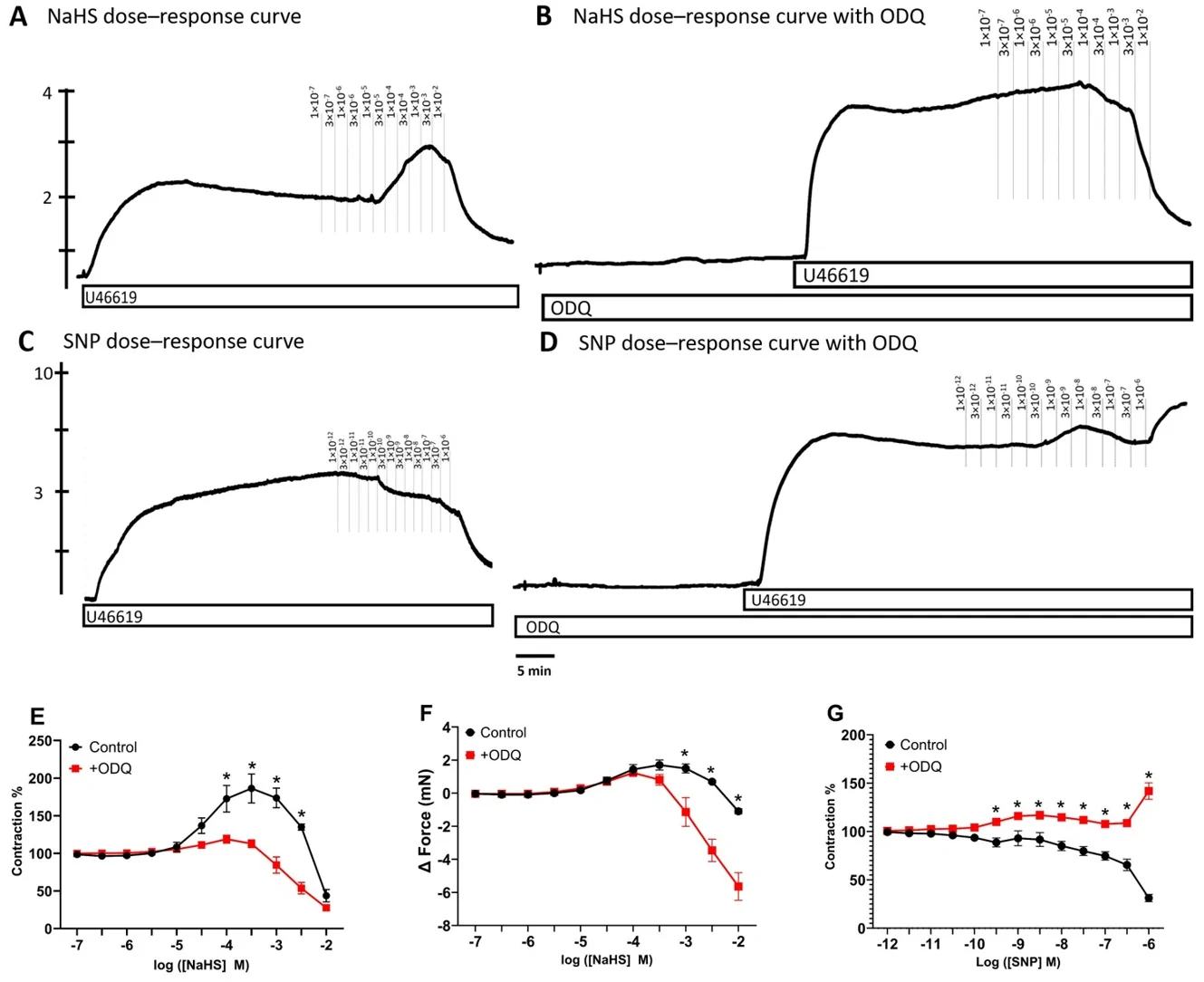
Effects of soluble guanylate cyclase inhibition on NaHS- and SNP-induced responses in rat intrapulmonary arteries. (**A**,**B**) NaHS and (**C**,**D**) SNP original traces of U46619-contracted rat intrapulmonary arteries showing responses to NaHS and SNP in the absence and presence of ODQ (3 × 10^−6^ M, 20 min). (**E**) NaHS and (**G**) SNP relaxation in rat intrapulmonary arteries expressed as a percentage of precontraction. (**F**) NaHS-induced force responses, where data are expressed as Δ force (mN), calculated as the force after each NaHS concentration minus the U46619-induced precontraction. The results are expressed as means ± SEM (n = 5–6). Two-way ANOVA was used to evaluate the effects of treatment and concentration, and Tukey’s post hoc test was used for comparisons: * *p* < 0.05 versus control arteries. Results of the two-way ANOVA analyses are presented in Supplementary Table S2.

Intrapulmonary arteries were incubated with a selective inhibitor of sGC, ODQ, to test the role of sGC in NaHS and SNP relaxation. Under control conditions, NaHS elicited a typical biphasic response, with vessels contracting more at lower concentrations and relaxing at higher concentrations. When sGC was inhibited by ODQ, the contractile effect of NaHS was reduced when expressed as a percentage of precontraction, whereas the relaxation phase remained similar to the control condition (Figure 4A,B,E). However, analysis of force changes showed that the contractile component was largely preserved, while the relaxant phase occurred at lower NaHS concentrations in the presence of ODQ (Figure 4F). To address the concern of increased U46619 contractions in the presence of ODQ, responses are additionally presented as absolute force values in Supplementary Figure S1. In contrast, when sGC was inhibited by ODQ, SNP-induced relaxation was abolished (Figure 4C,D,G), indicating that the NO released by SNP could no longer generate cGMP or activate protein kinase G.

### 3.4. Effects of H_2_S Synthesis Substrates on Vascular Relaxation

**Figure 5:**
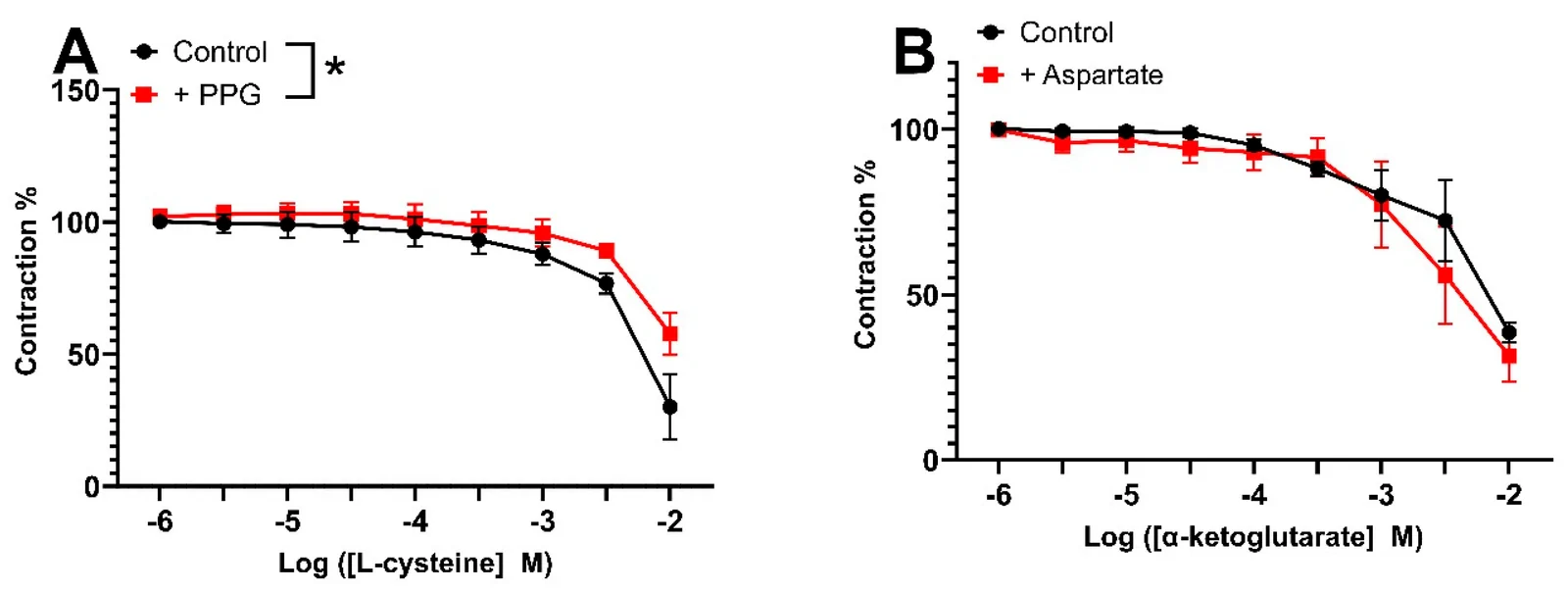
Effects of H_2_S synthesis substrates on relaxation of rat intrapulmonary arteries. (**A**) L-cysteine and (**B**) α-ketoglutarate relaxation in rat intrapulmonary arteries. U46619-contracted intrapulmonary arteries were incubated with L-cysteine or α-ketoglutarate in the absence or presence of PPG (10 mM, 30 min) (**A**) or aspartate (10 mM, 30 min) (**B**). The results are expressed as mean ± SEM (n = 5–6). Two-way ANOVA was used to evaluate the effects of treatment and concentration: * *p* < 0.05 versus control arteries. Results of the two-way ANOVA analyses are presented in Supplementary Table S2.

L-cysteine produced concentration-dependent relaxation in rat pulmonary arteries. Incubation with PPG significantly reduced this relaxation (Figure 5A), as indicated by a significant effect of treatment in two-way ANOVA (*p* = 0.0038), while there was no significant interaction between concentration and treatment. α-ketoglutarate produced concentration-dependent relaxation of pulmonary arteries. Incubation with aspartate did not affect these responses (Figure 5B).

### 3.5. Effects of Oxidative Stress on NO-Mediated Vascular Responses

**Figure 6:**
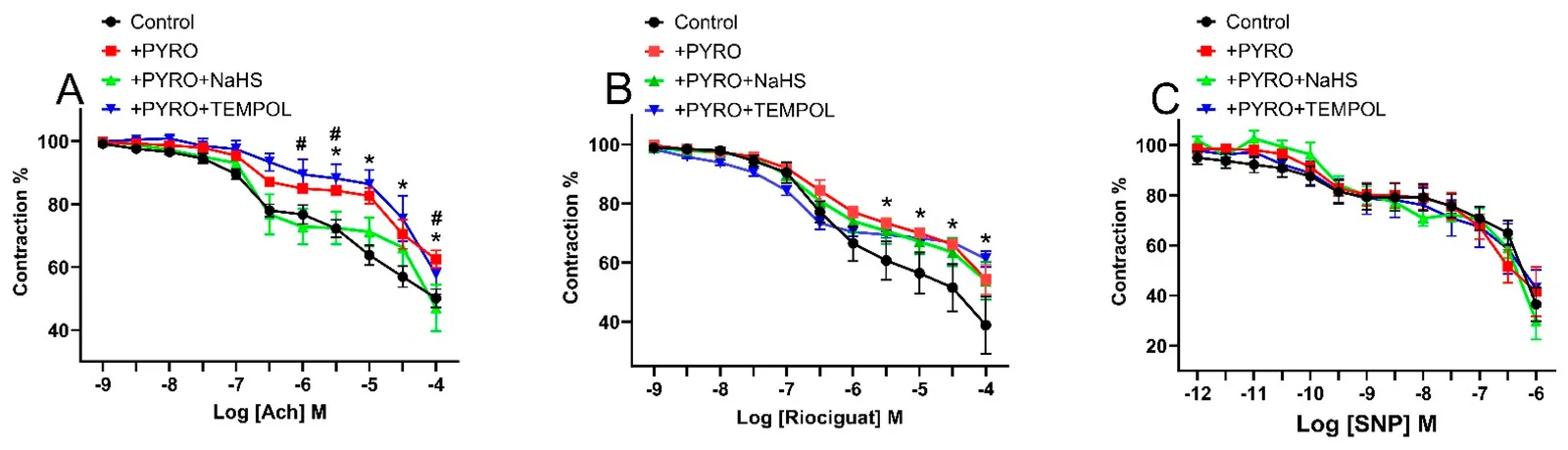
Effects of oxidative stress on ACh-, riociguat-, and SNP-induced relaxation in rat intrapulmonary arteries. (**A**) ACh, (**B**) riociguat and (**C**) SNP relaxation in rat intrapulmonary arteries. U46619-contracted intrapulmonary arteries incubated with pyrogallol and relaxed with ACh, riociguat or SNP, or incubated with pyrogallol (100 μM, 20 min) and NaHS (50 µM, 30 min) or tempol (100 μM, 20 min) and relaxed with ACh, riociguat or SNP. The results are expressed as mean ± SEM (n = 5–6). Two-way ANOVA was used to evaluate the effects of treatment and concentration, and Tukey’s post hoc test was used for comparisons: * *p* < 0.05, control versus +PYRO; # *p* < 0.05, +PYRO versus +PYRO+NaHS. Results of the two-way ANOVA analyses are presented in Supplementary Table S2.

Incubation of isolated rat intrapulmonary arteries with pyrogallol, a superoxide generator, resulted in a marked reduction in both endothelium-dependent relaxation and sGC-mediated relaxation. ACh-induced relaxation was significantly diminished following pyrogallol exposure (Figure 6A). Although riociguat directly stimulates sGC, its ability to induce relaxation was reduced after pyrogallol treatment, whereas SNP-induced relaxation remained largely unchanged (Figure 6B). These findings suggest that pyrogallol-induced oxidative stress impairs both endothelial NO availability and sGC function in pulmonary arterial smooth muscle. However, pyrogallol did not affect the SNP-induced response, and relaxation remained almost unchanged compared with the control group (Figure 6C).

The additional incubation with an H_2_S donor during pyrogallol exposure tended to improve ACh-induced relaxation (Figure 6A). Two-way ANOVA showed a significant interaction between concentration and treatment (*p* = 0.0496), as well as significant main effects of both concentration and treatment (*p* < 0.0001). Post hoc analysis showed that differences at higher ACh concentrations were mainly observed between control and pyrogallol-treated arteries and between control and NaHS-treated arteries, whereas no significant differences were detected at lower concentrations. In contrast, NaHS did not restore riociguat responses (Figure 6B), indicating that while H_2_S mitigates the decline in endothelial NO production or bioavailability by enhancing ACh responses, it does not completely reverse the oxidative stress-induced damage affecting sGC that compromises riociguat efficacy. Incubation with NaHS after pyrogallol did not affect SNP-induced relaxation (Figure 6C). Tempol was added after pyrogallol exposure, but it did not significantly change ACh, riociguat, or SNP-induced relaxation compared with pyrogallol alone. The lack of effect of tempol suggests that superoxide scavenging alone is insufficient to reverse the functional deficits that were induced by pyrogallol.

## 4. Discussion

An important strength of the present study is the simultaneous assessment of vascular force and intracellular Ca^2+^, which provided mechanistic insight beyond that obtained from pharmacological interventions alone. Although interactions between H_2_S and NO signaling have previously been described in systemic arteries, including rat mesenteric arteries and mouse aorta, much less is known regarding these interactions in resistance-sized pulmonary arteries. The present study demonstrates that endogenous H_2_S contributes to pulmonary vascular responsiveness to NO, whereas exogenous H_2_S relaxes pulmonary arteries largely through NO–sGC-independent mechanisms. Furthermore, oxidative stress differentially impairs endothelial and redox-sensitive sGC signaling while H_2_S partially restores endothelial function.

An important finding of the present study is that NaHS-induced relaxation occurred despite an increase in intracellular Ca^2+^. This observation indicates that the relaxant response cannot be explained simply by reduced intracellular Ca^2+^ and instead suggests a reduction in Ca^2+^ sensitivity or activation of downstream mechanisms regulating the contractile apparatus. This observation is consistent with our previous findings in rat mesenteric small arteries, where H_2_S-induced relaxation involved both reduced intracellular Ca^2+^ and Ca^2+^-independent mechanisms that led to force–Ca^2+^ uncoupling [19]. The present findings suggest that Ca^2+^-independent mechanisms may play an even more prominent role in H_2_S-induced relaxation of pulmonary arteries, although the underlying molecular pathways remain to be established.

The biphasic response to NaHS observed in rat intrapulmonary arteries resembles that previously described in rat mesenteric arteries [22,23]. We therefore examined whether NO–sGC signaling contributes to the balance between the contractile and relaxant components of this response. In the present study, L-NOARG altered the NaHS response in rat intrapulmonary arteries, suggesting the possibility that a similar mechanism may also be involved in this preparation. Likewise, ODQ-induced inhibition of sGC altered the biphasic response to NaHS. After correction for differences in U46619-induced precontraction, the effects of both L-NOARG and ODQ were most evident during the relaxant phase of the response, while the contractile component was mostly preserved. This is consistent with previous work in mouse aorta showing that H_2_S-induced vasoconstriction is absent in the aortae from eNOS−/− and sGCα1−/− mice and is not observed in vessels with the endothelium removed, highlighting the importance of endothelial NO-sGC signaling in this response [20]. Together, these findings suggest that endothelial NO-sGC signaling modulates the balance between the contractile and relaxant components of the NaHS response.

Our finding that inhibition of NOS or sGC abolished the low-concentration, NaHS-induced contraction suggests that basal NO–sGC signaling is required for this response. However, this does not imply that cGMP directly mediates H_2_S-induced contraction. One possible explanation is that reactions between NO and H_2_S generate reactive nitrogen–sulfur species, such as nitroxyl (HNO), thionitrous acid (HSNO), or nitrosopersulfide (SSNO^−^) [12], which have been proposed to modulate vascular function in rat mesenteric arteries [22]. Nevertheless, the present study did not investigate the formation of these species, and their contribution to pulmonary arterial contraction remains speculative. Alternative mechanisms involving modulation of NO signaling by endogenous H_2_S cannot be ruled out and warrant further investigation.

H_2_S also modulates ATP-sensitive K+ (K_ATP_) channel activity and is associated with PA relaxation [13]. Both in vitro or in vivo, H_2_S exerts antioxidant and anti-inflammatory effects and reduces pulmonary artery remodeling [14]. We found that inhibition of K_ATP_ channels with glibenclamide increased NaHS-induced contraction, indicating that K_ATP_ channels are involved in the H_2_S-induced vascular response. Since the blockade of K_ATP_ channels enhanced rather than reduced the contractile response, these channels are unlikely to be primary mediators of NaHS-induced contraction. Rather, the increase in contraction after K_ATP_ channel blockade indicates that these channels normally counteract, at least in part, H_2_S-induced contraction and may be involved in the relaxation observed at higher concentrations. A similar enhancement of H_2_S-induced vasoconstriction by glibenclamide has previously been reported in rat aorta, supporting the idea that K_ATP_ channel activation acts as a compensatory vasodilator mechanism that limits the contractile response [23]. These findings are supported by previous reports in systemic arteries showing that H_2_S activates K_ATP_ channels, leading to cell hyperpolarization and relaxation, particularly in smooth muscle, acting as an endogenous vasodilator and contributing to cardiovascular regulation [13]. At the molecular level, H_2_S has been proposed to activate K_ATP_ channels by directly modifying cysteine residues within the channel complex. Electrophysiological studies show that H_2_S increases K_ATP_ currents and channel open probability in vascular smooth muscle cells, while modification of cysteine residues in the sulfonylurea receptor subunit and persulfidation of K_ATP_ channel proteins have been implicated in this effect [24,25,26]. Proposed mechanisms include persulfidation of channel proteins and cleavage of disulfide bonds, which may alter channel conformation and promote channel opening [25]. These mechanisms may contribute to the K_ATP_-dependent component of the NaHS response observed in the present study. However, as channel activity and these molecular modifications were not measured directly, whether these mechanisms operate in rat intrapulmonary arteries remains to be determined. Loss or inhibition of K_ATP_ channel function promotes membrane depolarization, vasoconstriction, and smooth muscle proliferation in PH, whereas K_ATP_ channel openers (such as Iptakalim) reduce pulmonary pressure and vascular remodeling [27]. Thus, our findings reinforce the understanding of ATP-sensitive potassium channel participation in pulmonary vascular relaxation.

One of the principal findings of the present study was that inhibition of endogenous H_2_S synthesis attenuated SNP-induced relaxation. Because SNP releases NO directly and bypasses endothelial NO production, this observation suggests that endogenous H_2_S influences signaling downstream of NO generation. Recent studies in the corpus cavernosum microcirculation from CSE−/− mice have demonstrated that endogenous H_2_S regulates the redox state of sGC and helps maintain the enzyme in its reduced NO-responsive form [21]. Loss of endogenous H_2_S was associated with impaired NO-mediated relaxation and reduced responsiveness of the sGC pathway [21]. Therefore, a plausible explanation for the inhibitory effect of PPG on SNP-induced relaxation is that reduced endogenous H_2_S availability decreases the capacity to maintain sGC in its NO-sensitive state, thereby attenuating smooth muscle responses to NO released from SNP. This interpretation is consistent with our finding that PPG reduced relaxation induced by both ACh and SNP. While the effect on ACh responses could reflect reduced endothelial NO signaling, the attenuation of SNP-induced relaxation points to an additional action at the level of vascular smooth muscle. Previous studies in systemic arteries have mainly focused on the role of H_2_S-induced modification of eNOS and cIMP-dependent signaling in systemic arteries mediating H_2_S-induced contraction [22,28]. Our findings extend these observations by suggesting that endogenous H_2_S may also regulate the responsiveness of the NO-sGC pathway itself. Although we did not directly measure the sGC redox state, cGMP formation, or cIMP signaling, the data are consistent with a model in which endogenous H_2_S acts as a permissive factor that preserves NO-sGC signaling.

The enhanced SNP responses observed after inhibition of NOS and cyclooxygenase may reflect the removal of endogenous vasoconstrictor influences that normally oppose NO-mediated relaxation, thereby increasing sensitivity to exogenous NO. Under these conditions, PPG continued to inhibit SNP-induced relaxation, further supporting the notion that endogenous H_2_S modulates vascular smooth muscle responsiveness to NO independently of endothelial NO production. By contrast, the absence of an additional effect of PPG on ACh responses in the presence of L-NOARG and indomethacin suggests that the contribution of endogenous H_2_S becomes less apparent when both NOS- and COX-dependent pathways are blocked. Together, these findings are consistent with functional interactions between endogenous H_2_S and endothelial vasoactive mediators, although the precise molecular mechanisms remain to be established.

The distinction between endogenous and exogenous H_2_S in this study refers primarily to its source. Endogenous H_2_S is produced enzymatically within the vascular wall, whereas exogenous H_2_S was supplied as NaHS. This distinction is not absolute at the cellular level, as H_2_S readily crosses biological membranes and can act on neighboring cells [29]. Thus, H_2_S generated in the endothelium may also influence adjacent vascular smooth muscle. In pulmonary arteries, the H_2_S-producing enzymes are distributed across the vascular wall. CSE, CBS and 3-MST have all been reported in endothelial and smooth muscle cells, although their relative expression may vary depending on the species and vascular tissue or segment [14]. Endogenous H_2_S acting on pulmonary vascular smooth muscle may therefore originate from both the endothelium and smooth muscle itself. The different effects observed with endogenous and exogenously supplied H_2_S may reflect differences in where and how H_2_S is generated rather than the involvement of completely separate targets. Endogenous H_2_S is produced locally within vascular cells and can therefore act in close proximity to intracellular signaling proteins. By contrast, sulfide salts such as NaHS release H_2_S rapidly after dissolution, producing a different temporal and concentration profile from enzymatic H_2_S generation [30,31]. Moreover, the concentration of NaHS added to the organ bath does not necessarily reflect the amount of biologically available H_2_S reaching endothelial and smooth muscle cells. These differences in local availability and exposure may contribute to the different vascular responses observed in the present study.

In contrast to endogenous H_2_S, relaxation induced by exogenous H_2_S was preserved after inhibition of NOS and sGC, suggesting that NaHS-induced relaxation is mediated through mechanisms downstream or independent of the NO-sGC pathway. Activation of potassium channels, particularly KATP channels, likely contributes to these responses [22]. This interpretation is supported by the increase in NaHS-induced contraction after glibenclamide treatment. Furthermore, the reduction in L-cysteine-induced relaxation by PPG confirms a functional role for CSE-derived H_2_S in pulmonary artery relaxation, whereas the lack of effect of aspartate on α-ketoglutarate-induced responses suggests a lesser contribution of the CAT/3-MST pathway.

SNP-induced relaxation was abolished in the presence of ODQ, confirming that it depends on the NO–sGC–cGMP pathway. In contrast, NaHS-induced relaxation was preserved, indicating that H_2_S-induced relaxation is largely independent of sGC signaling. This suggests that NaHS-induced vasodilatation at higher concentrations occurs via cGMP-independent mechanisms, such as K_ATP or BK_Ca channels or other redox-sensitive pathways [19]. In addition, ODQ modified the biphasic response to NaHS. Although relaxation remained intact, the response shifted toward relaxation after sGC inhibition, suggesting that basal NO-sGC signaling influences how pulmonary arteries respond to H_2_S.

In addition, it was recently demonstrated that carbonyl sulfide (COS)/H_2_S-donor hybrid sGC stimulators can activate sGC in vitro and reduce fibrosis in transforming growth factor beta 1 (TGF-β1)-treated cardiac fibroblasts by increasing cGMP and H_2_S levels and improving cardiac function in heart failure by reducing myocardial fibrosis [32]. 

Oxidative stress impaired endothelium-dependent relaxation in pulmonary arteries, whereas SNP-induced relaxation was largely preserved. The reduction in ACh-induced relaxation is consistent with decreased endothelial NO bioavailability caused by the rapid reaction between superoxide and NO, thereby limiting NO diffusion to vascular smooth muscle. In contrast, the preservation of SNP-induced responses suggests that the downstream smooth muscle machinery required for NO-mediated relaxation remained largely functional under the experimental conditions employed. The partial restoration of ACh-induced relaxation by NaHS further supports the notion that H_2_S can preserve endothelial function during oxidative stress, possibly through antioxidant actions that improve NO bioavailability or limit oxidative damage to endothelial signaling pathways.

An important observation was that pyrogallol reduced riociguat-induced relaxation while leaving SNP-induced relaxation largely unchanged. This differential effect suggests that oxidative stress affects specific components of the NO–sGC pathway rather than causing a generalized impairment of vascular smooth muscle relaxation. Previous studies in the corpus cavernosum microcirculation from CSE−/− mice have demonstrated that the redox state of sGC is a critical determinant of its responsiveness to both endogenous and pharmacological stimuli [21]. A plausible explanation is that pyrogallol-induced oxidative stress promotes oxidation of the sGC heme environment or associated redox-sensitive signaling components, thereby reducing responsiveness to direct sGC stimulation. However, because SNP releases NO at pharmacological concentrations, NO-mediated activation of the remaining functional sGC pool may still be sufficient to preserve relaxation responses.

The inability of NaHS to restore riociguat-induced relaxation suggests that acute H_2_S administration does not fully reverse oxidative modifications affecting sGC signaling once established. Likewise, the lack of effect of tempol indicates that simple superoxide scavenging is insufficient to restore vascular responsiveness after pyrogallol exposure. Together, these findings suggest that oxidative stress induces more persistent alterations within the NO–sGC pathway, potentially involving oxidation of redox-sensitive protein targets, changes in sGC functionality, or downstream signaling mechanisms. Although these processes were not examined directly, the present data support a model in which oxidative stress impairs endothelial NO bioavailability and selectively alters redox-sensitive sGC signaling, while preserving the ability of exogenous NO to induce relaxation.

A limitation of the present study is that the vascular effects of exogenous H_2_S were not examined during pharmacological inhibition of endogenous H_2_S synthesis. Such experiments could further clarify whether endogenous H_2_S availability modifies vascular responses to exogenously administered H_2_S. Therefore, the distinction between endogenous and exogenous H_2_S made in the present study refers to the different experimental manipulations and functional responses observed, rather than to completely independent signaling pathways. In addition, sGC activity and cGMP levels were not measured directly. Consequently, the involvement of sGC signaling is inferred from pharmacological interventions rather than biochemical measurements. In addition, ODQ increased precontraction levels compared with control vessels (Table S1). Therefore, NaHS responses were also analyzed as changes in force relative to precontraction.

**Figure 7:**
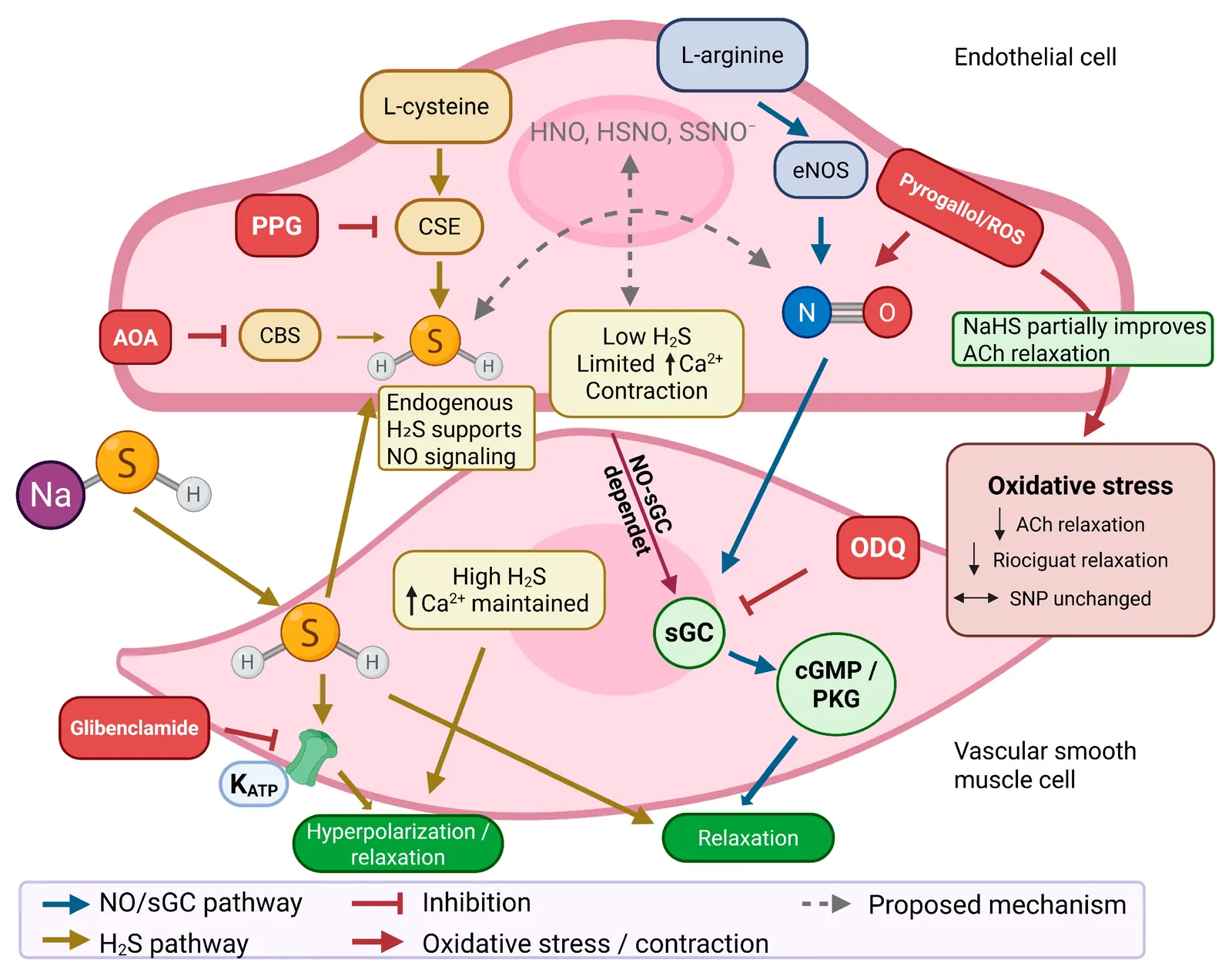
Proposed interactions between H_2_S and NO signaling in rat intrapulmonary arteries. Created in BioRender by Tunaityte, A., in collaboration with Volkevičiūtė, A. (2026) https://BioRender.com/67yjlo9.

Based on the findings of this study, Figure 7 presents a schematic overview of the proposed interactions between H_2_S and NO signaling in rat intrapulmonary arteries.

## 5. Conclusions

The present study demonstrates that hydrogen sulfide exerts complex, concentration-dependent effects on rat intrapulmonary arteries. Simultaneous measurements of vascular force and intracellular Ca^2+^ showed that NaHS-induced relaxation occurred despite maintained or increasing intracellular Ca^2+^, indicating that relaxation cannot be explained solely by reductions in intracellular Ca^2+^ and is consistent with the involvement of Ca^2+^-independent mechanisms. Inhibition of endogenous H_2_S synthesis reduced vascular responsiveness to exogenous NO, whereas exogenous H_2_S-induced relaxation was largely preserved following inhibition of NO synthase or soluble guanylate cyclase, supporting different functional roles of endogenous and exogenous H_2_S in regulating pulmonary vascular tone. Furthermore, oxidative stress selectively impaired endothelial function and responsiveness to riociguat, while NaHS partially restored endothelium-dependent relaxation. Collectively, these findings provide new mechanistic insight into the role of H_2_S in regulating pulmonary vascular tone and highlight the distinct contributions of endogenous and exogenous H_2_S to pulmonary vascular function under physiological and oxidative stress conditions.

## Data Availability

The original contributions presented in this study are included in the article/Supplementary Materials. Further inquiries can be directed to the corresponding author.

## Supplementary Materials

The following supporting information can be downloaded at https://www.mdpi.com/article/10.3390/biomedicines14092043/s1, Table S1: U46619-induced precontraction levels in rat intrapulmonary arteries under different experimental conditions. Table S2: Results of two-way ANOVA for concentration–response curves. Figure S1: Absolute force responses to NaHS in control and ODQ-treated intrapulmonary arteries.

## Abbreviations

The following abbreviations are used in this manuscript:

ACh	Acetylcholine
ANOVA	Analysis of variance
AOA	Aminooxyacetic acid
cGMP	Cyclic guanosine monophosphate
CBS	Cystathionine β-synthase
COS	Carbonyl sulfide
CSE	Cystathionine γ-lyase
DMSO	Dimethyl sulfoxide
eNOS	Endothelial nitric oxide synthase
ET-1	Endothelin-1
ETAR	Endothelin type A receptor
H_2_S	Hydrogen sulfide
HPV	Hypoxic pulmonary vasoconstriction
KPSS	Potasium-rich physiological saline solution
L-NOARG	Nω-nitro-L-arginine
MST (3-MST)	3-Mercaptopyruvate sulfurtransferase
NaHS	Sodium hydrosulfide
NO	Nitric oxide
ODQ	1H-[1,2,4]oxadiazolo[4,3-a]quinoxalin-1-one
PA	Pulmonary artery
PASMCs	Pulmonary artery smooth muscle cells
PH	Pulmonary hypertension
PPG	Propargylglycine
ROS	Reactive oxygen species
SEM	Standard error of the mean
sGC	Soluble guanylate cyclase
SNP	Sodium nitroprusside
TGF-β1	Transforming growth factor beta 1
VSMCs	Vascular smooth muscle cells
